# Polyubiquitin chain assembly and organization determine the dynamics of protein activation and degradation

**DOI:** 10.3389/fphys.2014.00004

**Published:** 2014-01-24

**Authors:** Lan K. Nguyen, Maciej Dobrzyński, Dirk Fey, Boris N. Kholodenko

**Affiliations:** ^1^Systems Biology Ireland, University College DublinDublin, Ireland; ^2^Conway Institute, University College DublinDublin, Ireland; ^3^School of Medicine and Medical Science, University College DublinDublin, Ireland

**Keywords:** ubiquitin, polyubiquitin chain, ubiquitination dynamics, bistability, oscillations, protein degradation, protein lifetime

## Abstract

Protein degradation via ubiquitination is a major proteolytic mechanism in cells. Once a protein is destined for degradation, it is tagged by multiple ubiquitin (Ub) molecules. The synthesized polyubiquitin chains can be recognized by the 26S proteosome where proteins are degraded. These chains form through multiple ubiquitination cycles that are similar to multi-site phosphorylation cycles. As kinases and phosphatases, two opposing enzymes (E3 ligases and deubiquitinases DUBs) catalyze (de)ubiquitination cycles. Although multi-ubiquitination cycles are fundamental mechanisms of controlling protein concentrations within a cell, their dynamics have never been explored. Here, we fill this knowledge gap. We show that under permissive physiological conditions, the formation of polyubiquitin chain of length greater than two and subsequent degradation of the ubiquitinated protein, which is balanced by protein synthesis, can display bistable, switch-like responses. Interestingly, the occurrence of bistability becomes pronounced, as the chain grows, giving rise to “all-or-none” regulation at the protein levels. We give predictions of protein distributions under bistable regime awaiting experimental verification. Importantly, we show for the first time that sustained oscillations can robustly arise in the process of formation of ubiquitin chain, largely due to the degradation of the target protein. This new feature is opposite to the properties of multi-site phosphorylation cycles, which are incapable of generating oscillation if the total abundance of interconverted protein forms is conserved. We derive structural and kinetic constraints for the emergence of oscillations, indicating that a competition between different substrate forms and the E3 and DUB is critical for oscillation. Our work provides the first detailed elucidation of the dynamical features brought about by different molecular setups of the polyubiquitin chain assembly process responsible for protein degradation.

## Introduction

Regulated degradation of proteins has pivotal roles in many cellular processes including cell cycle progression, transcription, signal transduction, differentiation, and apoptosis (King et al., 1996; Hershko and Ciechanover, 1998; Nguyen et al., 2011, 2013). Ubiquitination via the ubiquitin-proteosome system (UPS) is the primary degradation pathway through which the stability and levels of cellular proteins are controlled with high specificity. Its malfunctioning leads to a number of human diseases (Lonser et al., 2003; Guerriero and Brodsky, 2012; Sano and Reed, 2013). This is exemplified by the quality control endoplasmic reticulum (ER)-associated protein degradation (ERAD) pathway that targets misfolded or inappropriately accumulated proteins of the ER for ubiquitination and subsequent degradation by the 26S proteosome. Aberrant upregulation of ER stress induced ERAD can lead to apoptosis and ERAD dysfunction has been implicated in diseases (Guerriero and Brodsky, 2012; Sano and Reed, 2013). In yeast, supplying cells solely with the mutated form of ubiquitin (Lys48 to Arg) without the wild-type ubiquitin leads to cell-cycle arrest and a lethal phenotype (Finley et al., 1994). Disruption of the ubiquitination machinery, particularly the ligating enzymes, have also been implicated in the pathogenesis of several disorders including the Von Hippel-Lindau syndrome, Angelman syndrome and the autosomal-recessive growth retardation disorder 3-M syndrome (Lonser et al., 2003; Huber et al., 2005; Greer et al., 2010). Recent large-scale quantification of mammalian gene expression has revealed significant variability in the stability of thousands of cellular proteins with half-life ranging from a few minutes to a few months (Schwanhäusser et al., 2011), further highlighting the versatility of the UPS pathway.

**Figure 1:**
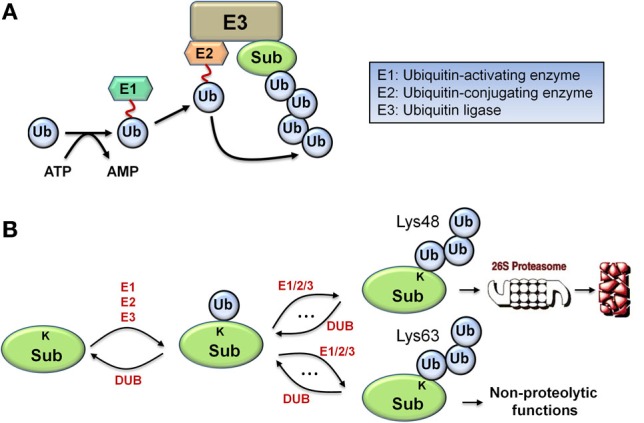
**Biological schematic diagrams of ubiquitination. (A)** Schematic diagram illustrating the sequential reaction steps of ubiquitin activation and transferring involving a cascade of E1/E2/E3 enzymes, leading to polyubiquitination of the target protein. **(B)** Simplified schematic diagram displaying the formation of two major types of polyubiquitin chain via repeated ubiquitination cycles: Lys48 and Lys63-linked chains, with differential functional consequences.

Monoubiquitination, the covalent tagging of a single ubiquitin molecule to the target substrate protein, is a multi-step process that involves a cascade of three essential enzymes: an E1 ubiquitin-activating enzyme, an E2 ubiquitin conjugating enzyme, and an E3 ubiquitin ligase (Figure 1A). The C-terminus of ubiquitin first forms a thioester bond with the catalytic systeine of the E1 in an ATP-dependent manner before being transferred from E1 to the catalytic systeine of the E2. Subsequently, the E3 ligase binds both the ubiquitin-charged E2 and the substrate to catalyze transfer of the ubiquitin to a lysine residue on the substrate to form an isopeptide bond, resulting in substrate monoubiquitination (Hershko and Ciechanover, 1998). Importantly, ubiquitin contains seven lysine residues which can be utilized by the appropriate E2/E3 pair to catalyze further cycles of ubiquitination, assembling various types of polyubiquitin chains. Polyubiquitination through ubiquitin Lys48 linkages generally targets the substrate protein for degradation, and is the principal focus of this work, while other linkages such as Lys63-linked chains are associated with non-proteolytic functions including kinase activation, signal transduction, and endocytosis, as illustrated in Figure 1B (Ye and Rape, 2009). Similarly to phosphorylation cycles, the action of the E3 ligase is reversed by a class of enzymes called deubiquitinases (DUBs) which catalyze the reverse deubiquitinating reaction.

A number of recent theoretical investigations have been carried out to explore the dynamic properties of signaling networks where ubiquitination and UPS-mediated degradation play major roles in their signal regulation (Nguyen et al., 2011, 2013). These studies have revealed that ubiquitination can bring about intricate dynamic behaviors including bistable switches, oscillatory and excitable responses to the system of interest. In these previous studies the assembly of polyubiquitin chains on target proteins were always considered as a single step. However, the chain assembly is in fact a dynamic and multi-reaction process. Here, we provide for the first time a detailed dynamic investigation of the assembly of polyubiquitin chain on a substrate protein mediated by multiple cycles of ubiquitination and subsequent degradation. Employing kinetic modeling, simulations and bifurcation analysis, we demonstrate that the formation of polyubiquitin chains is a highly dynamic process capable of generating complex temporal and steady-state behaviors. Using parametric and bifurcation analysis, we derive regions of distinct dynamics of the system. Further, we examine the effect of the ubiquitin chain length on the degradation dynamics and the steady-state distribution of the targeted protein's concentration. Applying the first-passage theory, we provide intuitive insights into the connection between protein concentration and average protein lifetime. Our results provide novel mechanistic insights into the intricate operation of the polyubiquitin chain assembly machinery.

## Results

### A core model of polyubiquitin chain assembly

#### Development of a core kinetic model

Polyubiquitin chain assembly is an essential marking mechanism for the recognition of a protein substrate destined for destruction. Previous studies have shown that the assembled chain must consist of at least four ubiquitin molecules for efficient proteasomal targeting (Thrower et al., 2000). Two competing models for the chain extension currently exist. The chain is either built in a stepwise manner with sequential addition of one ubiquitin monomer after another, or following an “en-bloc” transfer wherein the ubiquitin chain is pre-assembled on a E2 enzyme before being transferred to the substrate in a single step (Li et al., 2007; Ravid and Hochstrasser, 2007; Ye and Rape, 2009). Furthermore, a combined model where chains are formed following both mechanisms in a mixed manner can also exist.

**Figure 2:**
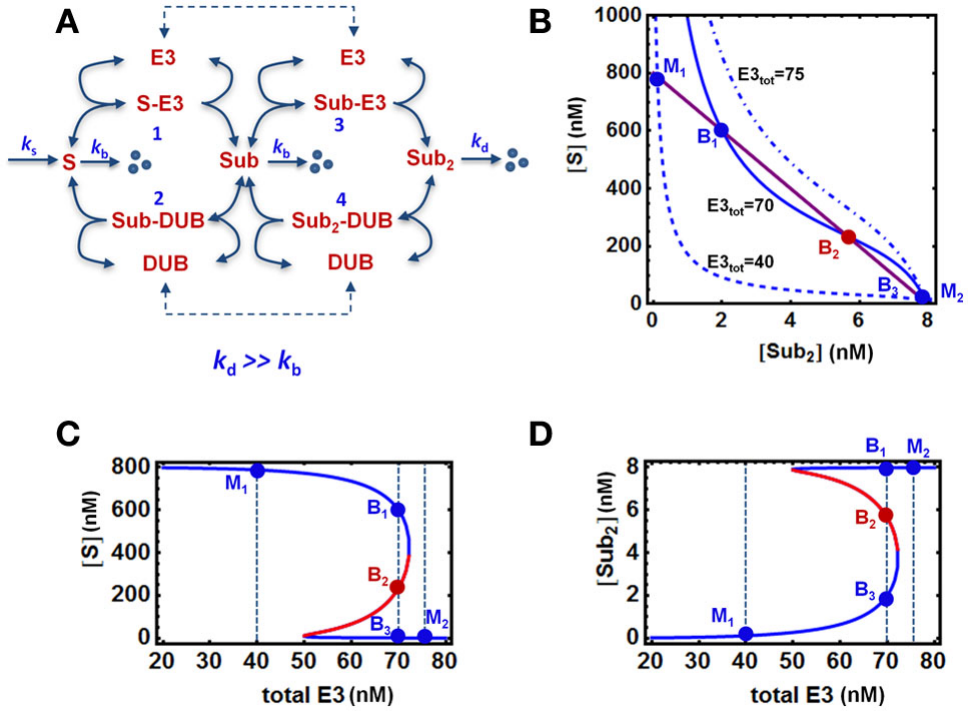
**Bistable, “all-or-none” responses in the core model. (A)** Mass-action kinetic scheme of a two-cycle chain formation system regulated by E3/DUB. The reactions and ordinary differential equations (ODEs) of the core model are given in Tables 1A,B. **(B)** Quasi steady state (QSS) curves intersection on the [S] vs. [S-ub_2_] plane determines the system steady states: 3 intersection points for bistability vs. 1 intersection point for monostability. **(C,D)** Bistable response and hysteresis curves of the system: dependence of steady-state levels of [S] and [S-ub_2_] on increasing abundance of the E3 ligase. The blue and red solid lines indicate stable and unstable steady states, respectively. The vertical dashed lines illustrate the systems steady states corresponding to three cases of E3 total: monostability (M_1_ and M_2_) and bistability (B_1–3_). Parameters values and units used for plotting are given in Table 1A, 4th column.

For simplicity, we fist consider a core model of polyubiquitin chain assembly consisting of only two ubiquitination cycles while longer cycles are examined in later sections. A schematic scheme describing the mechanistic interactions of this core model is given in Figure 2A. Here E3 ligase is assumed as a main catalyzing enzyme, regulating the dynamics of the chain formation in this model, while neglecting the presence of E1 and E2 enzymes. This assumption is supported by data suggesting that E2s, such as Cdc34, are mostly pre-charged with ubiquitin under steady-state condition (Jin et al., 2007). Importantly, the final ubiquitinated substrate with the longest assembled chain (S-ub_2_) is assumed to be subject to rapid degradation by the 26S proteosome, whereas the unmodified substrate or intermediate ubiquitinated substrate forms are under a basal, substantially lower degradation rate (*k*_*d*_ >> *k*_*b*_). The substrate degradation is balanced by a constant synthesis of the unmodified substrate S. We further assume that ubiquitin is in abundance (and not a limiting factor), therefore its concentration is considered a constant parameter in the core model. Based on this mechanistic reaction scheme, we constructed the model using ordinary differential equations (ODEs). We describe the (de)ubiquitination catalyzed by the E3 ligase and its opposing DUB enzyme at elementary step levels that follow mass-action kinetics which. Model reactions and ODEs are given in detail in Tables 1A,B.

**Table 1A:** **Reactions and reaction rates of the core, double-cycle model in Figure 2A**.

**No**	**Reactions**	**Reaction rates**	**Parameter values for Figure 2**	**Parameter values for Figure 4**
1	S + E3 ↔ S-E3	v_1_ = *k*_f1_·S·E3 – *k*_r1_· S-E3	*k*_f1_= 0.06, *k*_r1_= 0.1	*k*_f1_ = 0.002, *k*_r1_ = 0.7
2	S-E3 → E3+S-ub	v_2_ = *k*_c1_· S-E3	*k*_c1_ = 0.2	*k*_c1_ = 0.2
3	S-ub + DUB ↔ Sub-DUB	v_3_ = *k*_f2_· S-ub· DUB − *k*_r2_· Sub-DUB	*k*_f2_ = 0.01, *k*_r2_ = 0.1	*k*_f2_ = 0.01, *k*_r2_ = 0.006
4	Sub-DUB → DUB+S	v_4_ = *k*_c2_·Sub-DUB	*k*_c2_ = 0.2	*k*_c2_ = 0.25
5	S-ub + E3 ↔ Sub-E3	v_5_ = *k*_f3_· S-ub· E3 − *k*_r3_· Sub-E3	*k*_f3_ = 0.01, *k*_r3_ = 0.1	*k*_f3_ = 0.01, *k*_r3_ = 0.1
6	Sub-E3→E3+S-ub_2_	v_6_ = *k*_c3_·Sub-E3	*k*_c3_ = 4	*k*_c3_ = 4
7	S-ub_2_ + DUB ↔ Sub_2_-DUB	v_7_ = *k*_f4_·S-ub_2_·DUB – *k*_r4_·Sub_2_-DUB	*k*_f4_ = 0.01, *k*_r4_ = 0.1	*k*_f4_ = 0.06, *k*_r4_ = 0.043
8	Sub_2_-DUB → DUB+S-ub	v_8_ = *k*_c4_·Sub_2_-DUB	*k*_c4_ = 0.2	*k*_c4_ = 0.054
9	Ø → S	v_9_ = *k*_*s*_· S	*k*_*s*_ = 0.08	*k*_*s*_ = 0.08
10	S-ub_2_→ Ø	v_10_ = *k*_*d*_·S-ub_2_	*k*_*d*_ = 0.01	*k*_*d*_ = 0.01
11	S→ Ø	v_11_ = *k*_*b*_·S	*k*_*b*_ = 0.0001	*k*_*b*_ = 0.0001
12	S-ub→ Ø	v_12_ = *k*_*b*_·S-ub		

The first- (dissociation k_r_, catalytic k_c_, degradation k_d_, k_b_) and second-order (association k_f_) rate constants are expressed in s^−1^and nM^−1^ s^−1^. Synthesis rate is expressed in nMs^−1^

**Table 1B:** **Ordinary differential equations of the double-cycle model**.

**Left-hand sides**	**Right-hand sides**	**Initial concentrations (nM)**
d[S]/dt	v_9_ – v_1_ + v_4_ – v_11_	10
d[S-E3]/dt	v_1_ – v_2_	0
d[Sub-E3]/dt	v_5_ – v_6_	0
d[S-ub]/dt	v_2_ – v_5_ – v_3_+ v_8_ – v_12_	0
d[S-ub_2_]/dt	v_6_ – v_7_ – v_10_	0
d[Sub_2_-DUB]/dt	v_7_ – v_8_	0
d[Sub-DUB]/dt	v_3_ – v_4_	0
d[E3]/dt	– v_1_ + v_2_ – v_5_+ v_6_	100
d[DUB]/dt	– v_3_ + v_4_ – v_7_+ v_8_	100

*The reaction rates are given in Table 1A*.

Since polyubiquitin chain assembly can follow combined extension modes via stepwise or en-bloc transfer (Raman and Harper, 2009), chains with four ubiquitins can be formed from two ubiquitination cycles in several ways; e.g., by stepwise transfer followed by an en-bloc transfer of a three-ubiquitin chain, or two sequential en-bloc transfers of two-ubiquitin chains. This implies that our two-cycle core model described above is relevant for analysing the dynamics of chain assembly with length of four or even more ubiquitin monomers.

#### Key experimental observations of ubiquitination kinetics

Experiments aimed at obtaining kinetic insights and/or differentiating between competing models of ubiquitination are challenging due to the fast speed of ubiquitin-transfer reactions (Pierce et al., 2009; Raman and Harper, 2009). Nevertheless, a number of key experimental observations have been made which narrow our choice of the kinetic parameter values for the core model. Using the cyclin-dependent kinase inhibitor Sic1 as a substrate and its cognate E2/E3 pair (the cullin-RING ubiquitin ligase SCF^Cdc4^ and the ubiquitin-conjugating enzyme Cdc34), Petroski and Deshaies have examined the Lys48 polyubiquitination process (Petroski and Deshaies, 2005). They revealed that Sic1 polyubiquitination occurs in two distinct stages: addition of the first ubiquitin which is slow and rate limiting, followed by the attachment of subsequent ubiquitins at a much faster rate. As a result, Sic1 containing a single ubiquitin molecule (Sic1-ub) was ubiquitinated up to 5-fold faster than unmodified Sic1 (Petroski and Deshaies, 2005). This indicates that assuming the same DUB kinetics for the two ubiquitination cycles in the core model, the flux of the first E3-catalyzed ubiquitination cycle would be significantly lower than that of the second cycle. Moreover since SCF was found to increase the Vmax of the di-ubiquitin elongation reaction (Petroski and Deshaies, 2005), we can assume that the differential ubiquitination rate was primarily due to the lower catalytic rate (*k*_cat_) of the first cycle. More recent data lend further support to this observation (Pierce et al., 2009). Employing the same pair of E2/E3 but using cyclin E (CycE) and β-Catenin (β-Cat) as substrates, Pierce et al. performed mixing reactions on a millisecond timescale in a quench-flow device which allowed them to subsequently visualize the different substrate forms (Pierce et al., 2009). Fitting their data to a theoretical chain building model based on sequential transfers of ubiquitins, the authors could estimate the individual transfer and dissociation rates for each intermediate in the generation of polyubiquitinated CycE/β-Cat. Consistent with the previous results, they found that for both substrates once they are monoubiquitinated, the rate of chain elongation dramatically increases before slowing down again as the chain grows further. Interestingly, the substrate dissociation rate values are similar regardless of ubiquitin-chain lengths (Pierce et al., 2009). Overall, these data have provided useful qualitative knowledge for our model parameterization.

To further quantitatively parameterise the model, we estimated the core model kinetic parameters by equating the lifetime of the substrate between our model and Pierce et al's. Derived values for the E3-related parameters are given in Table 2. In the absence of well documented kinetic information on DUBs, we assume that DUBs operate on a similar timescale as the E3 ligases and DUB-related kinetic parameters have the same values for different ubiquitination cycles.

**Table 2:** **Model reactions, equations and kinetic parameter values for simplified models with varying chain length from 2 to 5 in Figures 5, 8**.

	**Reactions**	**Unit**	**1st cycle**	**2nd cycle**	**3rd cycle**	**4th cycle**	**5th cycle**
E3 catalyzed ubiquitination	S(-ub_*n*_) binding to E3 ligase	nM^−1^ s^−1^	0.01	0.01	0.01	0.01	0.01
	dissociation of S(-ub_*n*_)-E3 complex	s^−1^	0.1	0.1	0.1	0.1	0.1
	Catalysis	s^−1^	0.2	4	1	0.6	0.3
DUB catalyzed de-ubiquitination	S(-ub_*n*_) binding to DUB deubiquitinase	nM^−1^ s^−1^	0.01	0.01	0.01	0.01	0.01
	dissociation of S(-ub_*n*_)-DUB complex	s^−1^	0.1	0.1	0.1	0.1	0.1
	Catalysis	s^−1^	0.2	0.2	0.2	0.2	0.2
Substrate S	synthesis rate, *k*_*s*_	nM s^−1^	0.05	−	−	−	−
	degradation rate, *k*_*b*_	s^−1^	0.0002	0.0002	0.0002	0.0002	−
	degradation rate, *k*_*d*_	s^−1^	−	−	−	−	0.02

*The ODEs for the models of length 1–5 can be constructed straighforwardly from the schematic diagram in Figure 5A in a similar way as described in Tables 1A,B for model of length 2*.

### Bistable “all-or-none” switches in protein degradation

#### Emergence of bistable switches in the presence of degradation

We have previously reported the existence of bistable switches in a dual phosphorylation-dephosphorylation cycle with a distributive mechanism for the catalyzing kinase and phosphatase (Markevich et al., 2004). Bistability is a phenomenon in which a dynamic system switches between two distinct stable steady states but cannot rest in an intermediate state. Bistable behaviors have been observed in many biological systems and shown to be involved in cell-fate decision making and cell-cycle control (Bagowski and Ferrell, 2001; Xiong and Ferrell, 2003; Wang et al., 2009). A fundamental difference that distinguishes multiple phosphorylations from polyubiquitination (such as on Lys48) is the absence of rapid ubiquitination-triggered degradation. We thus explore if protein degradation would still give rise to bistable switches in the synthesis of polyubiquitin chains and if so, how would bistability be influenced by the length of the chain.

Analysis of the core model using parameter values derived in the previous sections show that abrupt, bistable switches can also emerge in the presence of rapid degradation of S-ub_2_ (Figure 2). The occurrence of bistability can be illustrated by plotting the nullclines (i.e., the solution curves for the ODE right hand sides wherethe time derivative is set to zero) on the systems phase plane. The intersection of the nullclines gives the equilibrium points of the ODE system. Strictly speaking, nullclines describe two-dimensional systems. In high-dimensional systems including our model, bistability can similarly be demonstrated by plotting the quasi steady-state (QSS) curves for two chosen variables (e.g., two substrate forms, S and S-ub_2_) on one plane where the system's steady state also correspond to intersection points of the curves. Figure 2B shows that depending on the total level of the E3 ligase, there can either be one or three intersection points between the QSS curves which correspond to monostable or bistable responses, respectively. At an intermediate level of E3 ligase, the QSS curves (solid lines) intersect at three points B_1−3_ resulting in bistability. The lower or higher total E3 shifts the QSS curve for S down (dashed) or up (dashdotted) on the plane, while leaving the S-ub_2_ QSS curve intact, resulting in just one intersection point (M_1_ or M_2_) and monostability.

As a consequence of bistability, hysteresis is observed for most system species, illustrated in Figures 2C,D for the unmodified substrate S and final ubiquitinated form S-ub_2_ in response to increasing E3 level (Figures 2C,D). The specific E3 total values selected for plotting in Figure 2B and the respective intersection points are shown on the hysteresis curves. As the E3 ligase abundance gradually increases from M_1_, the system starts at a high steady-state S (low steady-state S-ub_2_) and follows the high branch into the bistable regime before abruptly switching to a low S value (high S-ub_2_) at an E3 threshold value characterized by a saddle-node bifurcation. On other other hand, if the total E3 begins at a high level (M_2_) and gradually decreases, the system will traverse the low steady-state S (high S-ub_2_) branch into the bistable regime before abruptly switching to the high branch at a second, lower E3 threshold value defined by another saddle-node bifurcation point. Moreover, with E3 total within the bistable regime, either the low or high stable branch (B_1_ or B_3_) can be reached depending on the system's history.

#### Substrate production and degradation rates affect bistability

**Figure 3:**
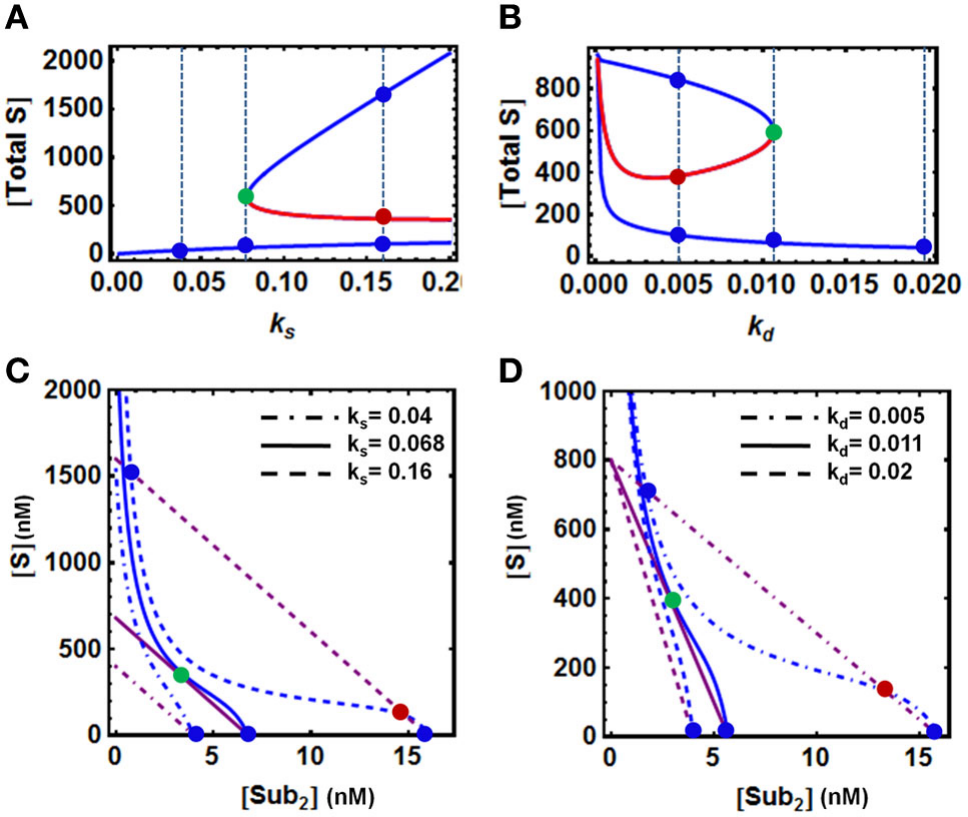
**Dependence of bistability on model parameters. (A,B)** Bistable response and hysteresis curves showing dependence of the steady-state levels of [S] and [S-ub_2_] on the rate of production (*k*_*s*_) and degradation (*k*_*d*_) of the substrate protein. The blue and red solid lines indicate stable and unstable steady states, respectively. The vertical dashed lines illustrate the systems steady states corresponding to three values of **(A)** *k*_*s*_ and **(B)** *k*_*d*_ displayed in **(C,D)**. **(C,D)** Corresponding QSS curves intersection determines the system steady states: three intersection points for bistability vs. 1 intersection point for monostability. The remaining parameters values and units used for plotting are given in Table 1A, 4th column.

To examine how the model parameters directly involved in protein level regulation affect bistability, we analyse the system behavior in response to change in the rate of production (*k*_*s*_) and degradation (*k*_*d*_) of the protein substrate. Summing up all the substrate forms (ubiquitinated and unubiquitinated) available in the model, we also observed hysteresis for the total S against *k*_*s*_ and *k*_*d*_. However, opposite changes occur when either *k*_*s*_ or *k*_*d*_ increase. (Figures 3A,B). Bistability was found over a wide range of the production rate, with the upper bound exceeding the normal physiological range, suggesting that sufficiently high production rate is necessary for bistable behavior (Figure 3A). On the other hand, although bistability persists over a large range of degradation rate, exceedingly strong degradation abolishes bistability (Figure 3B). The QSS plots for the corresponding various dynamics scenarios that are illustrated in Figures 3A,B are also shown in Figures 3C,D, where changes in the parameters *k*_*s*_ and *k*_*d*_ shift both QSS curves.

It should be noted that in the absence of any degradation (even with a very small basal rate for the unmodified substrate S, *k*_*b*_ = 0), under the specific conditions of low E3 ligase activity, the concentration of S can increase unlimitedly without reaching a steady state within a physiologically relevant timeframe. This is because the rate of S production would outweigh that of its maximal depletion rate. Although such case cannot be ruled out theoretically, it is highly unlikely to occur *in vivo*. Moreover, we found that if only the final substrate form, S-ub_2_ is degraded, a double (de)ubiquitination cycle cannot display bistability, as the final ubiquitinated form S-ub_2_ becomes independent of other variables at the steady state and always exhibits a single steady-state value. Thus, the inclusion of a basal degradation rate (which may be mediated by processes other than the UPS, e.g., the lysosome or other cellular proteases) for the intermediate forms of the substrate is necessary for the system to achieve bistable behavior.

#### Bistability for non-proteolytic polyubiquitination

Non-proteolytic polyubiquitination, exemplified by the Lys63-linked ubiquitin chain, has been known not to target the substrate for rapid degradation but instead direct other cellular functions. In this case, the chain assembly process would be an un-leaking, so-called “closed” system which resembles multi-site phosphorylation. Similarly to Lys48 polyubiquitination, bistability can also emerge in this system [data not shown, as this system is mathematically equivalent to a multisite phosphorylation system previously shown to exhibit bistability (Markevich et al., 2004)].

### Emergence of sustained oscillations due to rapid degradation

#### Emergence of sustained oscillations in the core model

**Figure 4:**
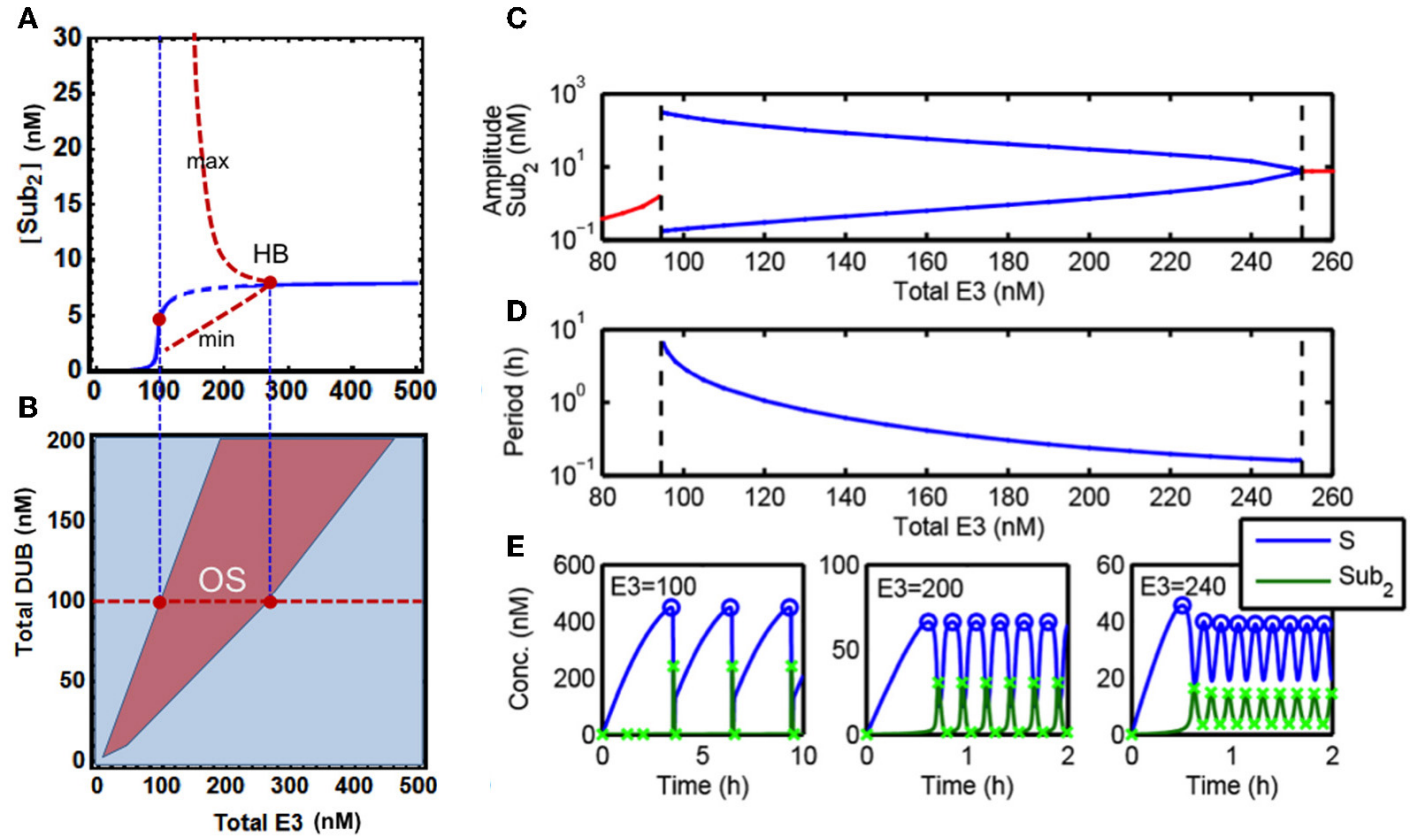
**Sustained oscillations in the core model. (A)** Bifurcation diagram of steady-state [S-ub_2_] concentration (blue line) vs. total E3 level showing existence of oscillatory dynamics in the core model. Dashed blue indicates the region of oscillations, and the red dashed lines indicate the minimum and maximum value of the oscillatory dynamics. **(B)** Two-parameter partition of system dynamics projected onto the E3-DUB plane. **(C)** Dependence of the oscillation amplitude (in log scale) on the total E3 level, blue lines indicate the minimum and maximum value of the oscillatory dynamics (as in **A**). **(D)** Dependence of the oscillation period (in log scale) on the total E3 level. The left and right dashed lines indicate the E3 values for which the oscillatory dynamics emerge and terminate. **(E)** Distinct temporal patterns of system species ranging from wave-like to pulse-like oscillatory dynamics resulted from different levels of the total E3 ligase. Parameters values used for plotting are given in Table 1A, 5th column. The x-marks indicate the maximum and minimum amplitude of the oscillations as used to generate **(A,C)**.

Despite the absence of any explicit negative feedback in our core model of chain assembly, we hypothesized that the existence of bistable behavior coupled with protein degradation which serves as a sink can bring about sustained oscillatory dynamics. We thus set out to examine this hypothesis. Comprehensive exploration of the parameter space, using systematic parameter sampling and visualization of the temporal dynamics, has enabled us to identify parameter domains where the system displays oscillations. Further analysis showed that robust oscillations can emerge within physiologically plausible parameter ranges (Figure 4).

Figure 4E shows the temporal oscillatory dynamics of S and S-ub_2_ at different levels of the total E3 ligase. The E3 level strongly influences the shape of oscillations, producing wave-like (with period in minutes) to distinct pulse-like pattern (period in hours) as in the case of S-ub_2_ concentration as the level of E3 decreases. Quantifying the oscillation amplitude and period revealed that both increase exponentially with decreasing E3 concentration until a threshold level is reached where the oscillations terminate abruptly (Figures 4C,D). To further characterize the oscillation dynamics, we carried out both local and global bifurcation analyses using Mathematica and XPPaut (Wolfram Research, 2010). A one-parameter bifurcation diagram in Figure 4A shows a stable limit cycle arising from the Hopf bifurcation accompanying an unstable fixed-point (dashed). This limit cycle suddenly disappears as the E3 concentration decreases below a specific value, denoted by the left red dot in Figure 4A. Furthermore, a two-parameter bifurcation plot can partition the E3-DUB plane into distinct dynamic regions, revealing a large oscillation regime indicative of the robust emergence of this behavior (Figure 4B).

It is important to point out that a sufficient degradation rate of the polyubiquitinated substrate is an essential condition for the observed oscillations. Our analysis shows that lack of degradation or even very small degradation rates prevent the oscillations. This suggests that oscillatory behavior is an intrinsic property of the “open” polyubiquitination-degradation system (i.e., the total abundance of interconverted protein forms is not conserved), that does not occur in “closed” systems such as multi-site phosphorylation which do not feature rapid degradation.

### Effects of chain length on systems dynamics

Although our two-cycle core model has provided novel insights into the dynamic behavior of ubiquitination chain extension, polyubiquitin chains are often made up of multiple ubiquitin monomers with length up to 10 (Pierce et al., 2009). Here, we investigate how both bistability and oscillations found in the two-cycle model are affected as chain elongation takes place.

#### Bistability is enhanced as polyubiquitin chain elongates

**Figure 5:**
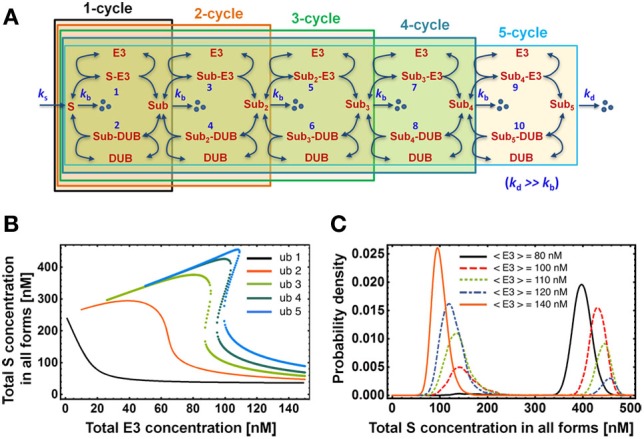
**Dependence of the dose response on chain length. (A)** Mass-action kinetic scheme of models consisting of 1 up to 5 ubiquitination cycles. Note that the reactions and ODEs for the models of length >3 are not explicitly given but can be easily constructed from the kinetic scheme following the format outlined in Tables 1A,B for length 2. **(B)** Dose-response curves for the total S concentration show the emergence of bistability for chains longer than 2 ubiquitination cycles. Dotted lines indicate the bistable region. **(C)** Steady-state distribution of total substrate concentration in all forms for the ubiquitination chain of length 5. Total E3 and DUB concentrations exhibit cell-to-cell variability modeled by gamma distribution with standard deviation equal to 10 nM. The histogram was obtained by solving numerically the system of corresponding ODEs for pairs of E3/DUB concentrations sampled 10,000 times from this distribution. Parameter values used for plotting are given in Table 2.

The increase in the number of ubiquitination cycles results in an increased non-linearity in the system and consequently in a steeper dose response against E3 ligase, which in turn enhances switch-like behavior of the system (Figure 5). This effect of the chain length on the dose response behavior is analogs to the result for a canonical MAPK cascade with multi-site phosphorylation cycles: each cascade layer increases steepness of the downstream response to external stimulation (Brown et al., 1997; Ferrell, 1997; Kholodenko et al., 1997). We find that for a biologically-derived parameter set (Table 2) the total substrate concentration indeed responds in an increasingly more switch-like fashion to changing E3 ligase levels in the presence of a longer ubiquitination chain. Moreover, owing to faster ubiquitination rate in the second cycle (*k*_cat_ = 4 s^−1^) compared to latter cycles (*k*_cat_ < 1 s^−1^), bistability emerges for ubiquitination chains longer than 2 (Figure 5B). The width of the bistable region increases only slightly for longer chains, which might indicate that the switch-like regulation of the substrate rather than the hysteretic response with wide bistable region is functionally relevant for cellular physiology.

Closely associated with steep switch-like response and bistability is the notion of a bimodal distribution of the protein level in a clonal population. In our system, each cell differs to some degree with respect to E3 and DUB concentration due to divergent gene expression profiles. Consequently, the overall population-level response consists of a superposition of slightly differing individual dose-response curves, which may give rise to a bimodal distribution of the substrate concentration. Such a response on the level of a cellular population may occur when the E3 enzyme level differs between cells within the ultrasensitive regime of the sigmoidal dose response (Birtwistle et al., 2012). Alternatively, bimodality may arise when E3 levels vary within the bistable region of the hysteretic dose-response (Becskei et al., 2001; Paliwal et al., 2007). We illustrate the latter in Figure 5C for the 5-cycle ubiquitination chain. Such population-level measurements with single-cell resolution can be performed with flow-cytometry or high content screening.

#### Relation between protein levels and average protein lifetime

Stochasticity inherent to all biochemical processes at the level of a single cell introduces variability in the protein lifetime. The lifetime τ of molecules undergoing simple one-step degradation follows an exponential distribution with the coefficient of variation (CV = SD / mean) equal 1 (Li and Qian, 2002). A sequential degradation process such as ubiquitination steps discussed here may result in a peaked, gamma-like lifetime distribution, which becomes narrower with an increasing number of steps (Dobrzyński, 2008; Pedraza and Paulsson, 2008; Shin et al., 2009). As a consequence the CV decreases too. In the simplest case of an irreversible reaction sequence with equal rate constants for each of the steps, the CV depends only on the number of steps *N* and follows CV = 1/($\sqrt{\text{N}}$) (Dobrzyński, 2008). This narrowing down of the lifetime distribution has been argued to bring about precision in biological processes: a much tighter regulation may take place when molecules exist in the cell for a duration narrowly distributed around the mean. For example, Li and Qian have demonstrated that GTP hydrolysis cycle modeled as four reversible steps significantly reduces the variance in the lifetime of the GTP-bound state (Li and Qian, 2002). Such an improved accuracy is arguably advantageous for precise signal amplification to downstream effectors. Due to very similar lifetimes (from a narrow distribution), each GTP-bound molecule activates an almost equal number of effectors. Signal relay is therefore robust every time the receptor signal triggers production of few GTP-bound molecules.

Peaked and narrow lifetime distribution also affects the decay pattern of the molecule (Dobrzyński, 2008). The analysis by Deneke et al. (2013) of experimentally measured mRNA decay time courses revealed deviation from the exponential decay and the existence of fast and slow decay patterns. These in turn have been attributed to sequential mRNA decapping that gives rise to non-exponential lifetime distributions. The effect of such distributions on the steady-state protein or mRNA variability is subtler, however. A seminal result from queuing theory states that for systems with exponentially distributed synthesis events, the lifetime distribution has no effect and the resulting variability of molecule level is Poissonian; such systems are known to be *insensitive* to the degradation (“service”) time distribution (Adan and Jacques, 2001). However, the result may rarely hold in a biological setting for the exponential timing of protein or mRNA synthesis often occurs in bursts, which strongly affect the stationary distribution (Shahrezaei and Swain, 2008).

We calculate protein lifetime distributions for various ubiquitination chain lengths shown in Figure 5A using first-passage theory (Redner, 2001). We conclude that the distributions are peaked but their CVs are close to 1, as in exponential distribution, and decrease weakly (less than 1%) with the addition of a single ubiquitination cycle (Figure 8). The multi-step degradation process is therefore close to memoryless and the protein lifetime distribution is well approximated by an exponential. As a consequence, the theoretical prediction follows that the decay pattern of a protein targeted for multi-step ubiquitination will undergo the classic exponential decay, for which the protein half-life equals Ln(2) × < τ > (angle brackets denote the average over all molecules of a given protein species within a cell, i.e., the average protein lifetime). Pulse-chase technique (Schwanhäusser et al., 2011) or light activated fluorescent tag such as Dendra2 (Zhang et al., 2007) can be used to measure decay dynamics.

Regardless of the time distribution of synthesis and degradation events, the average protein lifetime, < τ >, can be calculated by applying another general result of queuing theory, the Little's Law (Elgart et al., 2010). According to the theorem, the average lifetime is related to the average total protein level < S > and the average protein synthesis rate λ_*S*_ through the relation < S > = λ_*S*_ < τ >. A useful ramification of this relation is that any of the three quantities can be calculated by measuring the remaining two. More importantly, the rule holds regardless of the statistics governing protein production and degradation. In addition, the Little's rule implies that the bistability evidenced by the hysteretic dose-response in total S concentration shown in Figure 5B exists also in the average protein lifetime—the observation corroborated by the calculation of lifetime distributions shown in Figure 8B, where two lifetime distributions emerge that correspond to two stable protein level states.

### Bistability and oscillations persist in extended models

#### Development of the detailed E1/E2/E3 model

**Figure 6:**
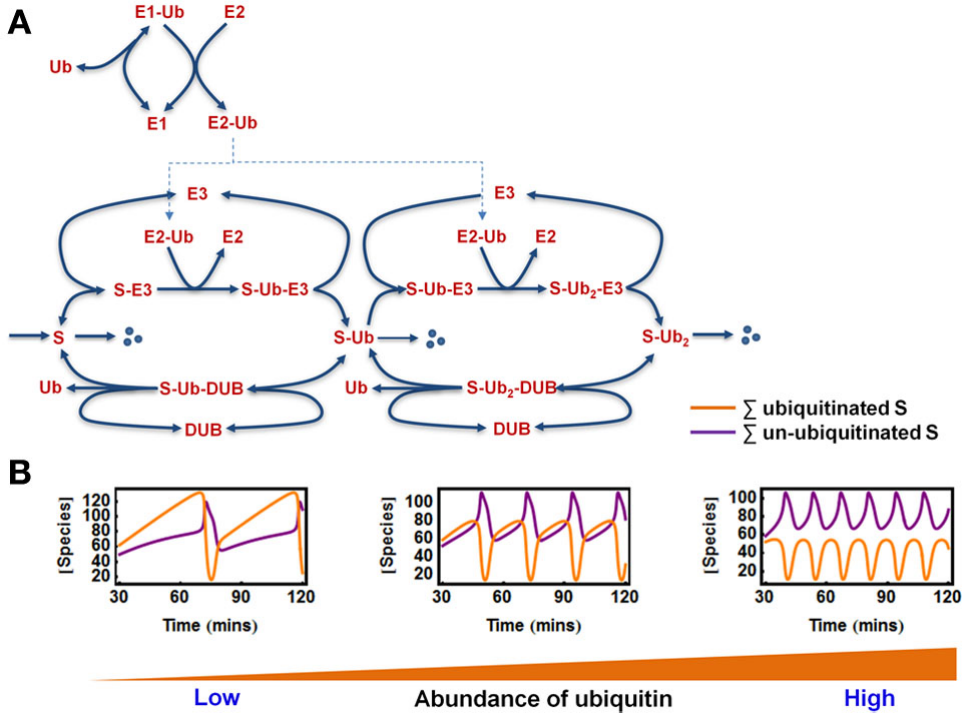
**Oscillations in the extended E1/E2/E3 model. (A)** Kinetic diagram showing the detailed model of polyubiquitin chain formation of length 2, with explicit incorporation of ubiquitin (Ub) and the E1, E2 enzymes. The reactions and ODEs of the this model are given in Tables 3A,B. **(B)** Dependence of oscillatory patterns on the abundance of available ubiquitin ranging from low to high level in the system where Ub is conserved. Parameters values and units used for plotting are given in Table 3A.

As discussed earlier, ubiquitination is a multi-step process involving not only the ligases but also the E1 activating enzyme, the E2 conjugating enzymes and the availability of ubiquitin molecule itself (Figure 1). It is thus important to see whether complex behaviors such as bistable and oscillatory responses still persist when potential effects exerted by the E1/E2 enzymes and ubiquitin status are explicitly accounted for. To this end, we extended the core model to incorporate the action of E1, E2, and ubiquitin within a detailed mechanistic model of two-cycle ubiquitin chain assembly, whose kinetic scheme is shown in Figure 6A. The model scheme follows largely the biological sequence of reactions described at a mechanistic level in Figure 1. A number of key assumptions were made.

First, we assumed that ubiquitin (Ub) becomes activated when bound to E1, and then it is loaded onto the E2 enzyme. The E2~Ub complex then binds to the pre-assembled S-E3 complex and transfers Ub to the substrate, releasing free E2. The E3 ligase can also dissociate from the complex yielding the free substrate in ubiquitinated form. The whole cycle repeats when the second Ub is conjugated to the mono-ubiquitinated substrate or when further Ub molecules are attached. Similar to the core model, the substrate is constantly produced and subjects to a low basal degradation rate while the end-product S-ub_2_ is rapidly degraded by the 26S proteosome. Furthermore, ubiquitinated substrates are continuously deubiquitinated by the DUB resulting in Ub recycling. Implied by the name, ubiquitin is ubiquitously expressed in most eukaryotic tissues and is a stable protein due to its unique globular structure (Shabek and Ciechanover, 2010). The *in vivo* pool of ubiquitin is therefore likely to be maintained at homeostasis under normal conditions by the dynamic equilibrium between multiple processes of Ub degradation and Ub synthesis. However, such pool can be altered in specific pathological contexts (Shabek and Ciechanover, 2010). In light of literature evidence, we thus consider two relevant scenarios: (1) when Ub is conserved in the system and (2) when Ub can be degraded together with the ubiquitinated substrate (Shabek et al., 2009) in which case the Ub pool is balanced by a constant synthesis of Ub (Figure 6A). The model reactions, ODEs and parameter values for both scenarios are given in Tables 3A,B.

**Table 3A:** **Reactions and reaction rates of the two-cycle extended model in Figure 6A**.

**No**.	**Reactions**	**Reaction rates**	**Parameter values**
1	S + E3 ↔ S-E3	v_1_ = *k*_f1_·S·E3 – *k*_r1_·S-E3	*k*_f1_ = 0.01, *k*_r1_ = 0.1
2	S-E3 + E2-ub→ E2 + Sub-E3	v_2_ = *k*_a1_ · S-E3·E2-ub	*k*_a1_ = 0.00003
3	Sub-E3 → E3 + S-ub	v_3_ = k_c1_· Sub-E3	*k*_c1_ = 0.2
4	S-ub + DUB ↔ Sub-DUB	v_4_ = *k*_f2_· S-ub·DUB – *k*_r2_·Sub-DUB	*k*_f2_ = 1.5, *k*_r2_ = 0.15
5	Sub-DUB → DUB + S + ub	v_5_ = *k*_c2_·Sub-DUB	*k*_c2_ = 1
6	S-ub + E3 → Sub-E3	v_6_ = *k*_f3_· S-ub· E3	*k*_f3_ = 1
7	Sub-E3 + E2-ub→ E2 + Sub_2_-E3	v_7_ = *k*_a3_·Sub-E3·E2-ub	*k*_a3_ = 0.01
8	Sub_2_-E3 → E3 + S-ub_2_	v_8_ = *k*_c3_·Sub_2_-E3	*k*_c3_ = 4
9	S-ub_2_ + DUB ↔ Sub_2_-DUB	v_9_ = *k*_f4_·S-ub_2_·DUB – *k*_r4_·Sub_2_-DUB	*k*_f4_ = 1.5, *k*_r4_ = 0.15
10	Sub_2_-DUB → DUB + S-ub + ub	v_10_ = *k*_c4_·Sub_2_-DUB	*k*_c4_ = 0.04
11	E1 + ub ↔ E1-ub	v_11_ = *k*_f5_·E1·ub – *k*_r5_·E1-ub	*k*_f5_ = 0.1, *k*_r5_ = 0.06
12	E1-ub + E2→ E1 + E2-ub	v_12_ = k_6_·E1-ub·E2	*k*_6_ = 0.004
13	Ø → S	v_13_ = *k*_*s*_·S	*k*_*s*_ = 0.06
14	Ø → ub (^*^)	v_14_ = *k*_*s*2_·S	*k*_*s*2_ = 0.001 *k*_*s*2_ = 0 (^*^)
15	S-ub_2_ → ub + ub S-ub_2_ → Ø (^*^)	v_15_ = *k*_*d*_·S-ub_2_	*k*_*d*_ = 0.05
16	S→ Ø	v_16_ = *k*_*b*_·S	*k*_*b*_ = 0.0001
17	S-ub→ Ø	v_17_ = *k*_*b*_·S-ub	

*The first-order (dissociation k_r_, catalytic k_c_, degradation k_d_, k_b_) and second-order (association k_f_) rate constants are expressed in s^−1^ and nM^−1^ s^−1^. Synthesis rate is expressed in nM s^−1^*.

* *Note that for the model where Ub is degraded with substrate, reaction 14 is absent (k_s2_ =*0*), and in reaction 15 Ub is not recycled*.

**Table 3B:** **Ordinary differential equations of the two-cycle extended model**.

**Left-hand sides**	**Right-hand sides**	**Initial concentrations (nM)**
d[S]/dt	v_13_ – v_16_ + v_5_ – v_1_	10
d[S-E3]/dt	v_1_ – v_2_	0
d[Sub-E3]/dt	v_2_ – v_3_	0
d[S-ub]/dt	v_3_ – v_4_ – v_6_+ v_10_ – v_17_	0
d[Sub_2_-E3]/dt	v_7_ – v_8_	0
d[S-ub_2_]/dt	v_8_ – v_9_ – v_15_	0
d[Sub_2_-DUB]/dt	v_9_ – v_10_	0
d[Sub-DUB]/dt	v_4_ – v_5_	0
d[E3]/dt	– v_1_ + v_3_ – v_6_+ v_8_	100
d[DUB]/dt	– v_4_ + v_5_ – v_9_+ v_10_	100
d[E1]/dt	– v_11_ + v_12_	1000
d[E1-ub]/dt	v_11_ – v_12_	0
d[E2]/dt	– v_12_ + v_2_ + v_7_	1000
d[E2-ub]/dt	– v_12_ – v_2_ – v_7_	0
d[ub]/dt (^*^)	v_14_ – v_11_ + v_9_ + v_10_ + 2 v_15_	10,000

*The reaction rates are given in Table 3A*.

* Note that for the model where ubiquitin is conserved, equation (^*^) is not included.

#### Oscillations and bistability in the extended model

We found that sustained oscillations persist in this more realistic model of chain building. Interestingly, the abundance of ubiquitin was found to strongly control the existence of oscillations and the oscillatory pattern (Figure 6B). In the case where Ub level is conserved, oscillations only arise when sufficient Ub is available. However, there appears to be no upper threshold of the Ub pool for the existence of oscillations, suggesting more abundant ubiquitin facilitates this dynamics. Moreover, higher Ub level is associated with lower oscillation period (Figure 6B). Similar to what was observed in the core model system, we also found that oscillations can only occur within defined (bounded) ranges of the E3 and DUB total levels. Similar observations were made for the E1 and E2 abundances, implying that appropriate levels of these key enzymes are necessary for the oscillation to arise.

**Figure 7:**
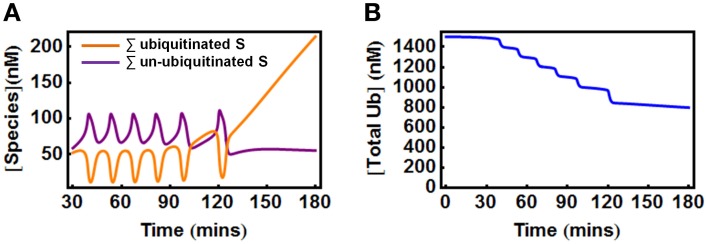
**Oscillations in the extended E1/E2/E3 model when Ub is degraded with the substrate. (A)** Oscillations vanish at about 120 min when the total Ub is depleted below a certain level due to its degradation, as shown in **(B)**. Parameters values and units used for plotting are given in Table 3A.

In the second scenario where Ub can be degraded by the 26S proteosome together with the substrate, the total Ub availability is a dynamic variable. Nevertheless, model analysis showed that to maintain oscillations, the Ub level should also be properly supplied as too low Ub abundance eliminates oscillation. Figure 7A shows an initial temporal oscillation of model species which subsequently vanishes when the level of the depleting Ub pool becomes lower than the threshold required for oscillations (Figure 7B). Increasing the Ub production rate can rescue oscillations by compensating for the level of lost Ub.

Furthermore, bistable behavior also exhibits in the two-cycle extended models in both cases when ubiquitin is conserved or being continually turned over. Consistent with the results from the core model, the bistable regime occurs over a defined range of E3 and DUB total and is also sensitive to the substrate production and degradation rates. We further extended the model to include up to four ubiquitination cycles. Importantly, having more ubiquitination cycles as a result of chain elongation does not appear to compromise the existence of both bistability and oscillations. Taken together, the results here indicate that oscillations and bistability are still inherent to the polyubiquitin chain formation process even when the effects of other enzymes than the E3 ligase are explicitly incorporated.

## Discussion and conclusion

### Competition of different substrate forms for the catalyzing enzymes is critical for complex dynamic behaviors

In the models of ubiquitin chain assembly presented above, substrate reactions catalyzed by the regulating enzymes E3 ligase and DUB are modeled explicitly using a mass-action description at the elementary step level. Under this kinetic formulation, the different forms of the target substrate compete for the catalyzing enzymes. For instance, the increased E3 portion bound in the first ubiquitination cycle will result in less E3 available for the second cycle. To examine if such competition should be a prerequisite for the emergence of oscillations and/or bistability, we modeled instead the (de)attachment of a single Ub in the core model with simple non-competitive Michaelis-Menten (MM) formulation, where the reaction rate implicitly contains the concentration of the catalysing enzyme [*V*_max_ = *k*_cat_^*^(Enzyme)]. Simple non-competitive MM description of both E3 and DUB-regulated reactions failed to lead to oscillations or bistable switches. However, using a form of competitive MM description based on total quasi-state approximation described in our previous work (Markevich et al., 2004), bistability and oscillations can both be found. These results together indicate that enzyme competition is critical for the existence of oscillations and bistability, as such competitions coupled with proper level of non-linearity would endow the system with effect of positive or negative feedback which underlie bistable response and oscillations, respectively.

### Supporting experimental evidence of switch-like ubiquitination

The theoretical predictions of potentially bistable and oscillatory behavior await future experimental validation. The field of ubiquitination, ubiquitin-related signaling and ubiquitin-associated detection techniques is maturing with rapidly accelerating pace. The studies of the epidermal growth factor receptor (EGFR)-activated signal transduction (where ubiquitination is strongly involved in signaling regulation (Nguyen et al., 2013), have shown that in response to graded levels of the EGF ligand, EGFR ubiquitination follows a switch-like, threshold-characterized response (Sigismund et al., 2013). This observations were verified by the measurements of ubiquitinated EGFR using both ELISA and stable isotope labeling with amino acids in cell culture (SILAC)-based mass-spectrometry. Importantly, the switch-like dose-response of the ubiquitinated EGFR correlates exactly with the EGFR internalization mediated by non-clathrin endocytosis, a primary mechanism responsible for EGFR degradation (Sigismund et al., 2013).

*In vitro* experimental design based on the model analysis results could also be the next step in confirming the predictions about the dynamics of the systems components. A reconstituted *in vitro* system with purified forms of relevant proteins (i.e., E3 ligase and DUBs) provides flexibility where one can explore wide ranges of enzyme concentrations. If compartmental localization is required, some of these involved proteins may be embedded into a phospholipid membrane bilayer or liposomes, which also can be utilized to facilitate complexes formation and enhance reactions rates. Potential temporal oscillatory behavior can be explored by adding the substrate protein followed by the addition of ubiquitin, the E1/E2 enzymes, E3 ligase and DUB to the reaction medium. At periodically selected time points, aliquots are taken, and the ubiquitinated level of the substrate can be measured by immunoblotting using relevant antibodies. Specific antibodies raised against common types of linkage including Lys48 and Lys63 are now available. More direct *in vivo* approaches such as imaging techniques using microscopy-based binding assay can also be exploited for high temporal resolution measurement [105], which have been previously used to detect oscillations in GTPases network (Carlin et al., 2011). Rapid development of quantitative mass-spectrometry based methods for measuring ubiquitinated protein level in bulk would be another viable option (Sigismund et al., 2013). In these *in vivo* setups, one may choose to inhibit the 26S proteosome to prevent UPS-mediated degradation, and therefore better identify the ubiquitination signals. On the other hand, detection of switches can be done by similar measurement techniques in response to increasing titration of a dose component, for example the regulating E3 ligase. Single-cell based approaches such as flow-cytometry or high content screening can serve an ideal platform for detecting the bimodal distribution of protein levels, indicative of the existence of switches or bistable responses.

### Implications of switch-like protein degradation regulation

There are multiple accounts of switch-like regulation that have been demonstrated experimentally for biological systems. Cascaded signaling networks exhibit steep dose responses that can translate analog input signal into all-or-none response of the transcription factor (Brown et al., 1997). Likewise, cooperative binding of transcription factors can give rise to steep relation between the factor concentration and the promoter activity (Rossi et al., 2000; Veitia, 2003). Here, we suggest an additional layer of digital regulation at the level of protein degradation due to a sequence of ubiquitination cycles. Our theoretical considerations demonstrate that for proteins degraded by way of multi-step polyubiquitination, steep or even hysteretic dose responses may arise when changing the parameters, such as the synthesis and degradation rates or the total ligase and deubiquitinase concentrations. Hence, a number of properties beneficial for cellular physiology can be argued. For example, even without pronounced steepness in the dose response at the signaling level or without strong cooperativity at the stage of transcription initiation, the protein level can still be regulated in a switch-like manner in response to intra- or extra-cellular cues. Such a switch-like dependence may also facilitate a filter to temporal fluctuations in the rate of protein synthesis introduced by transcriptional and translational bursting or pausing. Furthermore, resistance to fluctuations extends to changes in the concentration of the ligase and deubiquitinase (Figure 5B). Due to the steep sigmoidal relation between total substrate S and E3/DUB, large variations in the latter translate to digital responses in S. Interestingly, for the biologically-relevant parameters we find that the switch-like response is enhanced in the presence of longer ubiquitination chains. Notably, only proteins tagged with at least four ubiquitins are targeted by the proteasome, which strengthens our conjecture that switch-like regulation of protein degradation resulting from bistability may indeed be functionally utilized by cells to facilitate cellular decisions. Since there are less than 1000 different types of E3 ligase and even far less for E2 and E1 enzymes (Ye and Rape, 2009), a common E3 ligase is likely to be shared by multiple target substrates. In this case, a degradation regulation system enabled by multiple switch-like dose-response curves with different E3 threshold values for each substrate could be a possible mechanism to generate substrate specificity.

### Concluding remark

Since the discovery of protein ubiquitination more than three decades ago, subsequent work has revolutionized our perception of its role in signaling networks. As a principal mechanism for specific cellular protein degradation controlling many key cellular processes, polyubiquitination exhibits overwhelming diversity and complexity whose functional properties only begin to be unraveled. Our work contributes to this endeavor by providing a comprehensive investigation into the dynamic features of the process of ubiquitin chain formation.

## Materials and methods

### Construction of the kinetic models

To construct the kinetic models analyzed in section Results which includes: (1) the simplified, double-cycle ubiquitination model used in section A core model of polyubiquitin chain assembly, Bistable “all-or-none” switches in protein degradation, and Emergence of sustained oscillations due to rapid degradation (2) the simplified models with increasing chain length (up to 5 cycles) used in section Effects of chain length on systems dynamics and (3) and the detailed E1/E2/E3 model used in section Bistability and oscillations persist in extended models, we employed a deterministic modeling approach based on ordinary differential equations. The reaction rates were formulated at the elementary step level using mass-action kinetic law. Detailed reactions, reaction rates and the ODEs as well as parameter values for the models are given in the following tables. Specifically, the simplified, double-cycle model can be reconstructed from Tables 1A,B; the simplified models with varying chain length can be reconstructed from Table 2 and the detailed E1/E2/E3 model can be constructed from Tables 3A,B.

### Estimation of kinetic parameters for sequential ubiquitination cycles

The lack of experimentally measured kinetic data remains a general challenge for computational modeling. For our models of sequential ubiquitination cycles, the choice of kinetic parameters has been guided by key experimental observations wherever available or typical values for protein association/dissociation and enzymatic reaction rates, as explained in section Key experimental observations of ubiquitination kinetics. In particular, we estimated the kinetic parameters for the simplified models of various lengths, given in Table 2, based on *in vitro* measurement by Pierce et al. (2009). We assume that the rate of catalysis, *k*_*c*(*n*)_ in our model, corresponds to the ubiquitin transfer rate by Cdc34-SCF E3 ligase. We set complex dissociation rate, *k*_*r*(*n*)_ in our model, to 0.1 s^−1^, which is of the order of the dissociation rate *k*_off_ measured by Pierce et al.

### Simulation and bifurcation analysis procedure

Models implementation, temporal simulations, and calculation of the QSS curves were conducted in *Mathematica*. QSS curves were calculated as previously described (Nguyen et al., 2011). Bifurcation diagrams (including saddle-node and Hopf bifurcation) and the dependencies of steady states on parameters were calculated by numerical continuation algorithms using the software XPPAUT (www.math.pitt.edu/~bard/xpp/xpp.html).

### Calculation of protein lifetime distributions using first-passage theory

After synthesis, a single substrate molecule S undergoes a sequence of biochemical reactions such as formation of an intermediate complex with E3 ligase or deubiquitnase, and addition of ubiquitin tags (Figure 5A). In our model, the lifetime of S is the time between the synthesis and the degradation through either of the channels with rate constant *k*_*b*_ or *k*_*d*_. These reactions are of stochastic nature due to molecular fluctuations within the cell, hence the lifetime of S will follow a distribution rather than a single fixed number. In order to calculate that distribution we resort to first-passage time theory (FPT) (Redner, 2001).

First, we note that the ODEs that describe the dynamics of our system (e.g., Tables 1B or 3B) correspond to the set of chemical master equations for subsequent states of substrate S modifications. The rates of transitions between the states are described by reaction rate constants multiplied by steady-state concentrations of relevant chemical species. For instance, the transition from state S to enzyme-bound S-E3 takes place with the rate *k*_f1_× [*S*−*E*3]. By taking the Laplace transform of both sides of the original time-dependent system, we obtain a set of algebraic equations, which we solve for all the state variables: S, Sub, S-E3, etc. The lifetime of S is the first time the single molecule S reaches any of the degradation states (signified by dots in Figure 5A) from the initial state [S] = 1. Using the example of a two-cycle ubiquitination, the FPT is obtained as a flux from all the states that may directly lead to the degradation. In the Laplace-transformed s-space, flux is:

$$f\left(s\right)=k_{b}\left(\left[S\right](s)+\left[Sub\right](s)\right)+k_{d}[Sub_{2}](s)$$

**Figure 8:**
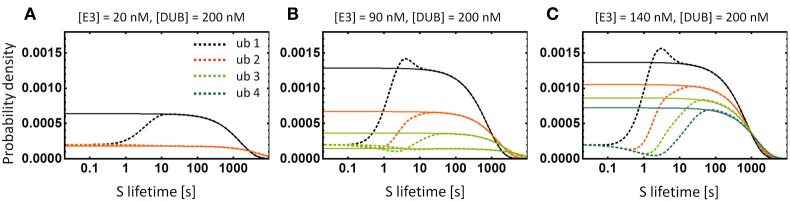
**Lifetime distributions of the substrate protein S. (A–C)** Dashed lines show the numerically calculated distributions of S for the models depicted in Figure 5. Solid lines show the exponential distributions with the same average lifetime as the corresponding lifetime distribution. Note that the exponential function is a very good approximation and discrepancies occur only for short lifetimes. Bistability of the system with chains longer than 2 results in two lifetime distributions that correspond to two stable states of the system, shown in **(B)**. For clarity, we show the curve for four ubiquitination cycles only in **(C)**.

By Taylor-expanding *f(s)* we can calculate moments of the FPT (or protein lifetime) and obtain the mean or variance. The inverse Laplace transform will yield the time-dependent function which has the interpretation of the probability density function. We plot it for various lengths of the ubiquitination sequence in Figure 8.

### Conflict of interest statement

The authors declare that the research was conducted in the absence of any commercial or financial relationships that could be construed as a potential conflict of interest.
